# 多种超分辨荧光成像技术比较和进展评述

**DOI:** 10.3724/SP.J.1123.2021.06015

**Published:** 2021-10-08

**Authors:** Jie CHEN, Wenjuan LIU, Zhaochao XU

**Affiliations:** 1.中国科学院分离分析化学重点实验室, 中国科学院大连化学物理研究所, 辽宁 大连 116023; 1. CAS Key Laboratory of Separation Science for Analytical Chemistry, Dalian Institute of Chemical Physics, Chinese Academy of Sciences, Dalian 116023, China; 2.中国科学院大学, 北京 100049; 2. University of Chinese Academy of Sciences, Beijing 100049, China

**Keywords:** 超分辨荧光成像, 纳米尺度, 可视化, 活细胞结构和动态, super-resolution fluorescence imaging, nanometer scale, visualization, cellular structure and dynamics

## Abstract

所见即所得是生命科学研究的中心哲学,贯穿在不断认识单个分子、分子复合体、分子动态行为和整个分子网络的历程中。活的动态的分子才是有功能的,这决定了荧光显微成像在生命科学研究中成为不可替代的工具。但是当荧光成像聚焦到分子水平的时候,所见并不能给出想要得到的。这个障碍是由于受光学衍射极限的限制,荧光显微镜无法在衍射受限的空间内分辨出目标物。超分辨荧光成像技术突破衍射极限的限制,在纳米尺度至单分子水平可视化生物分子,以前所未有的时空分辨率研究活细胞结构和动态过程,已成为生命科学研究的有力工具,并逐渐应用到材料科学、催化反应过程和光刻等领域。超分辨成像技术原理不同,其具有的技术性能各异,限制了各自特定的技术特色和应用范围。目前主流的超分辨成像技术包括3种:结构光照明显微镜技术(structured illumination microscopy, SIM)、受激发射损耗显微技术(stimulated emission depletion, STED)和单分子定位成像技术(single molecule localization microscopy, SMLM)。这些显微镜采用不同的复杂技术,但是策略却是相同和简单的,即通过牺牲时间分辨率来提升衍射受限的空间内相邻两个发光点的空间分辨。该文通过对这3种技术的原理比较和在生物研究中的应用进展介绍,明确了不同超分辨成像技术的技术优势和适用的应用方向,以方便研究者在未来研究中做合理的选择。

## Abstract (en)

“Seeing is believing” is the central philosophy of life science research, which runs through the continuous understanding of individual molecules, molecular complexes, molecular dynamic behavior, and the entire molecular network. Living and dynamic molecules are functional in nature; therefore, fluorescence microscopy has emerged as an irreplaceable tool in life science research. However, when fluorescence imaging is performed at the molecular level, some artificial signals may lead to erroneous experimental results. This obstacle is due to the limitation of the optical diffraction limit, and the fluorescence microscope cannot distinguish the target in the diffraction-limited space. Super-resolution fluorescence imaging technology breaks through the diffraction limit, allows visualization of biomolecules at the nanometer scale to the single-molecule level, and allows us to study the structure and dynamic processes of living cells with unprecedented spatial and temporal resolution. It has become a powerful tool for life science research and is gradually being applied to material science, catalytic reaction processes, and photolithography as well. The principle of super-resolution imaging technologies is different; therefore, it has different technical performances, thus limiting their specific technical characteristics and application scope. Current mainstream super-resolution imaging technologies can be classified into three types: structured illumination microscopy (SIM), stimulated emission depletion (STED), and single-molecule localization microscopy (SMLM). These microscopes use different complex technologies, but the strategy is the same and simple, i.e. two adjacent luminous points in a diffraction-limited space can be spatially resolved by time resolution. SIM has been used for three-dimensional real-time imaging in multicellular organisms; however, compared with other technologies, its lower horizontal and vertical resolutions need to be further optimized. STED is limited by its small imaging field of view and high photobleaching; however, the best time resolution can be considered at a high spatial resolution, and it has been proven that three-color STED imaging can be performed. In SMLM super-resolution imaging, the time resolution is affected by the time required to locate all fluorophores, which is closely related to the switching and luminescence properties of the fluorophore. With the improvement in horizontal and vertical resolution of imaging, the image acquisition speed, photobleaching characteristics, and the possibility of multi-color and dynamic imaging have increasingly become the key determinants of super-resolution fluorescence imaging. Thus far, the main use of super-resolution imaging technology has been focused on biological applications for studying structural changes less than 200 nm in dimension. In addition to the combination of structural and morphological characterization with biomolecular detection and identification, super-resolution imaging technology is rapidly expanding into the fields of interaction mapping, multi-target detection, and real-time imaging. In the latter applications, super-resolution imaging technology is particularly advantageous because of more flexible sample staining, higher labeling efficiency, faster and simpler readings, and gentler sample preparation procedures. In this article, we compare the principles of these three technologies and introduce their application progress in biology. We expect the results described herein will help researchers clarify the technical advantages and applicable application directions of different super-resolution imaging technologies, thus facilitating researchers in making reasonable choices in future research.

所见即所得是生命科学研究的中心哲学,贯穿在不断认识单个分子、分子复合体、分子动态行为和整个分子网络的历程中。活的动态分子才是有功能的,这决定了荧光显微成像在生命科学研究中成为不可替代的工具。但是当荧光成像聚焦到分子水平的时候,所见并不能给出想要得到的。这个障碍是由于受光学衍射极限的限制,荧光显微镜无法在衍射受限的空间内分辨出目标物。为了克服衍射极限的限制,以Stefan Hell、William E. Moerner、Eric Betzig、Xiaowei Zhuang、Mats G. L. Gustafsson为代表的科学家从20世纪90年代开始寻求方法,最终在2008年前后使得超分辨成像技术趋于成熟^[1]^。Hell^[2]^发明了受激发射损耗显微技术(stimulated emission depletion, STED), Moerner^[3]^开创了单分子定位技术(single-molecule localization microscopy, SMLM), Betzig等^[4]^提出了单分子定位成像的原理,并发明了光激活定位显微成像技术(photoactivated localization microscopy, PALM), Zhuang等^[5]^发明了基于荧光小分子的随机光学重构超分辨成像技术(stochastically optical reconstruction microscopy, STORM), Gustafsson^[6]^发明了结构光照明显微镜技术(structured illumination microscopy, SIM)。这3种技术为主要代表的超分辨荧光成像技术已在生命科学和材料科学中展示出巨大的威力和应用前景。本文通过对这3种技术的原理比较和在生物研究中的应用进展介绍,期望帮助研究者明确不同超分辨成像技术的技术优势和适用的应用方向,以方便研究者在未来研究中做合理的选择。

## 1 超分辨成像原理

### 1.1 SIM:简单的样品制备及中等分辨率的提高

SIM和STED技术均可归于基于特殊强度分布的照明显微成像技术一类。SIM成像的原理如图1a所示^[7]^,在荧光显微成像中,通过对系统点扩散函数的卷积和对样品的荧光强度分布可获得样品的荧光图像,相当于对荧光强度的频谱进行了低通滤波。因而空间上相邻的荧光分子在密集的分子群中表现出高的空间频率,这些高频分量对应样品的细微结构,在通过物镜被低通滤波截断后不能被检测器接收,因而传统荧光成像时丢失了大量样品精细结构的信息,如何收集这些高频信息成为突破衍射极限的关键。为了达到这个目的,SIM技术利用了莫尔效应,莫尔效应是通过用结构化光照射样品而产生的,当两个周期性图案重叠时,会出现称为莫尔条纹的较粗的图案,该空间频率恰好偏移了结构化模式的频率。在莫尔效应的影响下,SIM将传统荧光显微镜丢失的高频信息移入到可被检测的低频区域,而后通过进一步的算法优化和处理将其中的高频信息进行恢复,从而提高了成像的分辨率^[8]^。值得注意的是,SIM在焦平面上的分辨率约为传统荧光显微镜的2倍,即约为100 nm^[9]^。这是由于SIM的照明模式是由两束激发光经过物镜后在样品面上干涉产生,因此周期性的条纹也是衍射受限的。

**图1:**
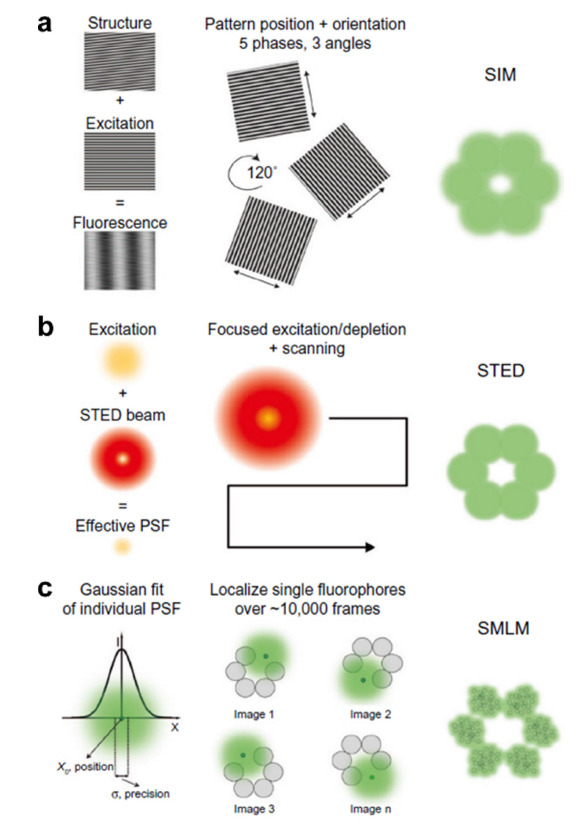
主要超分辨率技术原理

近些年SIM也出现了一些重要的改进技术,Gustafsson等^[6]^在2005年基于荧光团在强激光下的非线性效应提出了饱和结构光照明显微术(saturated structured illumination microscopy, SSIM),这一技术将传统SIM的横向分辨率提升到了约40 nm。但是这种成像技术也存在一定的缺点,SSIM的激发时间较长且激发功率较高,因此这种方法在应用于活细胞成像时受到了很大的局限。Sonnen等^[10]^在2008年开发了3D-SIM技术,将横向分辨率及纵向分辨率分别提升至约100 nm和约300 nm。但目前SIM作为一种基于宽场的超分辨显微成像技术仍存在一些问题。首先,基于宽场照明的激发模式的能量密度较低,因此在成像过程中很容易受到样品散射的影响,且其组织穿透深度较低,其应用大多局限在单细胞层面。SIM面临的另一个挑战是图像处理速度,特别是随着图像采集速度的迅速提高,如何足够快地处理这些数据变得越来越具有挑战性。此外,样品所引起的相差也是SIM在实验数据采集中或数据重构过程中需要加以解决或加以修正的问题^[11]^。

SIM具有样品制备简单,成像速度快,成像视野大等优势,目前已趋于成熟并已经实现了商业化,在生物学领域受到了广泛的应用。此外,与其他超分辨成像技术相比,SIM对于成像分辨率的提升并不依赖于特殊的荧光探针,普通的荧光团不需要特殊的性质就可以使用,可选择的荧光分子多,特别适用于活细胞多色动态成像。

### 1.2 STED:更高的分辨率

STED是第一种应用于细胞成像的远场超分辨成像技术,该技术通过纯光学方法实现对荧光团亮态和暗态的调控,利用受激辐射效应来压缩发光点的点扩散函数从而绕过光学衍射极限限制。

荧光分子在受到激发后由基态S_0_态跃迁到电子激发态振动能级 ${S_{n}}^{*}$ ,而后弛豫到激发态的最低振动能级S_1_态回到基态S_0_并发出荧光,这一过程称为自发辐射过程。但是如果荧光分子在激发态还未来得及向基态跃迁发射光子时系统内恰有第二个光子,且其能量恰好等于荧光团基态与激发态能级差,此时荧光团则会吸收第二个光子并同时发射两个与吸收的第二个光子具有相同能量、频率、相位及传播方向的光子,即发生受激辐射过程,自发辐射过程则被抑制^[12]^。STED建立在共聚焦显微镜基础上,采用两个激光器分别作为激发光光源和损耗光光源,其中损耗光光源产生的激光束通过螺旋相位板转变为中空的激光光束,并与激发光光源经过准直叠加后照射到样品上,二者共同作用产生缩小的自发辐射光斑,得到STED超分辨图像(见图1b)^[7]^。对于生物样品来说,STED的横向分辨率通常在30 nm到80 nm之间,具体取决于样品的性质和消耗激光的应用功率^[13]^。

在STED成像中要获得更高的分辨率就需要高效地使损耗光照射的荧光团进入暗态,自发辐射通常在样品被激发后几个纳秒内发生,因此需要严格控制受激辐射发生的时间,通常激发光要先于损耗光约200 ps到达样品,才可以保证荧光团被有效激发和损耗。此外,由于时间范围很短,且荧光分子的受激辐射横截面很小,要想完全耗尽重叠区域的荧光就需要非常高的STED光强度,STED光强度越大,系统的空间分辨率也就越高。从理论上说,无限增加耗尽激光功率可以无限提高STED的分辨率。但在实际应用中,损耗光功率过大容易导致细胞光毒性和荧光团的光漂白等问题,这是STED技术在活细胞成像中迫切需要解决的挑战^[11]^。

理论上所有的荧光发色团均能发生受激辐射以得到STED超分辨成像,但由于高强度损耗光(一般为共聚焦激发光的100倍)所造成的光漂白问题,使STED在应用的初期可用的染料范围相当有限。理想的STED染料应具有高亮度、高光稳定性的、大斯托克斯位移等特点,目前报道可用于STED成像的荧光探针主要可分为3类:荧光蛋白、有机小分子染料和荧光纳米颗粒。2006年Willig等^[14]^利用绿色荧光蛋白(GFP)实现了病毒和内质网的超分辨成像,其分辨率达到70 nm。但与有机小分子荧光染料和荧光纳米颗粒相比,荧光蛋白的光稳定性和亮度仍有较大的差距,目前依旧难以实现较长时间的STED成像。而一些结构优化过的有机染料分子被证明具有良好的抗光漂白性质,如结构优化后的硅罗丹明类染料可以在长达1500 s的STED成像后仍保留近70%的荧光信号^[15]^。目前也已经有多种商业化染料可供选择,如ATTO系列、Abberior系列等^[16]^。此外,一些高亮度抗漂白的荧光纳米颗粒,如量子点、上转换纳米颗粒、染料复合等离子体纳米颗粒等材料也正在被逐渐应用于STED成像领域^[17]^。

### 1.3 SMLM:单分子定位成像

除STED和SIM技术外,SMLM技术也是一种可以克服衍射极限而获得超分辨图像的成像方法。SMLM是基于单个荧光团在on态(荧光)和off态(非荧光)之间的随机切换,在特定的时间内,利用组合激光束(激活和激发激光束)照射使荧光团显示出荧光开关或闪烁,荧光团的随机光开关在每一帧图像中只产生少量的荧光点,在收集到足够的光子数后,通过二维高斯函数拟合的分析算法来帮助精确定位单个荧光团,最后通过合并所有单独激活的荧光团的位置重建超分辨图像,这种方法通常被认为是基于探针的单分子定位超分辨率显微镜^[18]^。该技术是通过数千帧荧光图像的重建来获得单幅高空间分辨率图像,这种技术中最常见的是光激活定位显微成像技术(PALM)和随机光学重构超分辨成像技术(STORM),这两种方法的机理如图1c所示^[7]^。

光激活荧光蛋白具有特殊的光开关特性,这类荧光蛋白仅有在被激活后才会被激发而发出荧光,Betzig等^[19]^在2006年首次利用这种具有光开关性质的绿色荧光蛋白实现超分辨PALM成像。首先用405 nm激活光照射目标区域激活少量的荧光蛋白,随后再用561 nm的激发光使被激活的荧光蛋白发出荧光,采集并定位这些单分子的位置直至荧光分子被漂白,重复“激活-激发-定位-漂白”的过程后将所有定位点叠加后重构得到超分辨图像。STORM的原理与PALM的原理类似,主要区别在于STORM使用的是光转换荧光染料(Cy3-Cy5荧光对)对样品进行标记,通过激光控制Cy5分子的“on-off”态从而获得单分子信号,通过重复“激活-激发-定位-漂白”的过程最终重构出超分辨图像。

与STED和SIM技术不同,单分子定位技术需要用特定的荧光探针标记样品,通过调控其开关特性从而将空间上重叠的多分子荧光图像在时间上进行分离,并得到一系列子图像,进而利用单分子定位算法精准定位荧光团后将图像进行叠加从而重构出超分辨图像。在这种花费时间分辨率来获得高分辨率的成像过程中,具有高亮度、高光稳定性、优异开关性质的荧光材料成为实现高分辨率SMLM成像的关键因素^[18]^。目前已经有一系列适用于SMLM成像的荧光材料被开发出来,其中具有光开光性质的荧光蛋白和有机小分子染料占据了主导地位。前述的具有光开关性质的绿色荧光蛋白是由野生GFP突变而得^[10]^,并最早被应用于SMLM成像,但其亮度较低导致成像的信噪比低,且最小采集时间约为150 ms/帧限制了其进一步生物应用^[11]^。随着进一步优化,目前已经有多种光激活荧光蛋白被开发,其发射波长可覆盖可见光区,为成像提供了丰富的选择。此外,具有更高亮度、更高光稳定性、更小尺寸的小分子光开关也获得了普遍关注,光开关类荧光分子(如Cy5、Alexa Fluor 647)和光笼类荧光分子(如Caged Q-rhodamine、Caged carboxyfluorescein)等已经被广泛应用于超分辨SMLM成像中^[20,21]^。

超分辨SMLM技术通过简单荧光“on-off”实现了在纳米尺度的超高精度的荧光成像。但是它的缺点也很显著,SMLM技术对于荧光团的准确定位来源于光子数的采集,又因为需要使荧光团稀疏发光的特点,因此需要采集大量的子图像进行数据分析与重构,计算量大且耗时很长,难以对细胞中的动态过程进行实时跟踪^[22]^。此外,SMLM技术的样品制备过程复杂且要求严格,包括反复照射导致的荧光团光漂白等问题使该技术在用于活体成像时仍有诸多问题亟待解决。

### 1.4 3种超分辨成像技术比较

超分辨荧光成像技术的出现对于生命科学的发展无疑有着举足轻重的意义,这3种成像技术基于不同的原理均打破了衍射极限的桎梏,有力地推动了生命科学的快速进步^[23]^。在进行活细胞成像时,空间分辨率和时间分辨率之间的制衡会影响研究者对于这3种超分辨成像技术的选择。SIM已经被证明可以用于多细胞生物中的三维实时成像,但与其他技术相比,其较低的横向及纵向分辨率均仍需进一步优化;STED尽管受限于成像视野小、高光漂白性的缺点,但在高空间分辨率下可兼顾最佳的时间分辨率,并且已经证明了可以进行三色STED成像;而在SMLM超分辨成像时,时间分辨率受定位所有荧光团所需的时间限制,具体与荧光团的开关、发光性质密切相关。随着成像的横向和纵向分辨率的提高,图像采集速度、光漂白特性及多色、动态成像的可能性也越来越成为超分辨荧光成像的关键决定因素。简而言之,这3种主要技术各有优缺点,选用哪种技术需取决于样品细节和所要研究的生物学问题^[7,11,24]^。

## 2 超分辨成像的生物应用

### 2.1 SIM

尽管相比于STED及SMLM, SIM对分辨率仅有中等程度的提高,但具有成像速度快、染料兼容性强、样品制备简单等突出优势,是一种特别适用于活细胞动态成像的超分辨成像技术。目前的研究已经证实,SIM在呈现活细胞中复杂结构之间的相互作用的研究中具有突出优势。

2007年,Schermelleh等^[25]^报道了3D-SIM用于动物细胞成像的一个典型案例(见图2a)。他们在多达3个通道中同时观察固定的小鼠C2C12成肌细胞的染色质、核层和核孔复合体。SIM突出的三维成像能力,使得与核层通道共定位的单个核孔复合体的分辨成为可能。此外,他们还能够区分核膜内外不同的孔复合蛋白。DNA结构中的空洞导致核孔复合体的产生,表明DNA被排除在这些连接细胞质和细胞核的孔之外。这一早期工作为三维分辨率倍增的宽场荧光显微镜、多色通道的同时成像以及使用传统荧光团标记感兴趣的细胞结构提供了一个令人印象深刻的案例。后期研究者通过与电子显微镜对比,对3D-SIM的有效性进行了验证^[26]^。

**图2:**
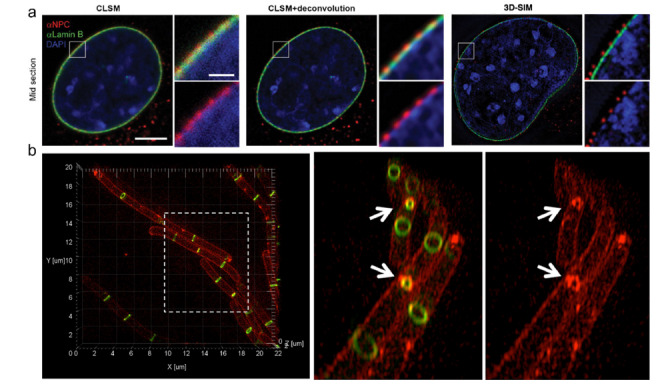
3D-SIM成像

通过成像和分析玉米减数分裂过程中染色体中的突触复合体发现,这些结构的侧部单元刚好低于常规光学显微镜可以分辨的极限。通过使用结合DNA的荧光团和荧光标记抗内聚蛋白REC 8*α*-kleisin同源物抗体,他们发现:1.这种蛋白的分布沿横向是不连续的;2. DNA的卷曲发生在突触复合体形成之后;3.这些复合物被卷曲成左手双螺旋结构。通过分析整个减数分裂过程中的这些结构,他们揭示了这些双螺旋结构在减数分裂的不同阶段的短暂性质。这项关于染色质结构的工作很快被其他3D-SIM研究跟进,他们分析了人类中心体在不同细胞周期(如有丝分裂期和有丝分裂间期)的性质。SIM荧光染色抗体的高特异性和SIM扩展的三维成像深度使其成为分析细胞内结构(例如,结构蛋白在所有维度上的共定位)的宝贵工具。

SIM在生物医学领域的应用也开始出现。在一个早期的例子中,Brown等^[27]^使用3D-SIM来确定皮质肌动蛋白在NK细胞(natural killer cell)突触附近的重构。这种紧密肌动蛋白网的重塑被认为为NK细胞创造了所需的空间,使其分泌裂解颗粒到目标细胞,从而杀死病毒感染或转化的细胞。此外,Cogger等^[28]^首次用荧光显微镜对肝窦内皮细胞中纳米孔的结构和分布进行了成像。由于在此之前这一结构只能用电子显微镜进行研究,而电子显微镜样品的制备需要严苛的程序,以至于有生物学家怀疑这些结构可能是样品制备过程中产生的污染。

微生物学是另一个率先引入SIM技术的领域。在该领域,目前集中在研究FtsZ的结构和分布上(见图2b)。FtsZ是真核细胞中微管蛋白的细菌同源物,它组装成的Z环是引起细菌细胞分裂的原因。Strauss等^[29]^使用3D-SIM对枯草芽孢杆菌和金黄色葡萄球菌成像时发现,在这两种菌类中Z环是不连续的;长时间动态成像进一步揭示了具有Z环的FtsZ位置是动态的,并且在整个细胞分裂过程中保持异质性。

SIM超分辨成像技术也被用于病毒结构的分析。Horsington等^[30]^使用3D-SIM对单个痘苗病毒颗粒进行成像,借助荧光成像来跟踪该病毒的成熟和形态的变化。3D-SIM的三维特性使他们能够将病毒包膜蛋白B5解析为围绕该病毒的球形结构,从而提供了判断病毒颗粒成熟程度的手段。

SIM超分辨成像适应于活细胞成像的关键是照明模式产生和样品扫描速度,目前已经利用电光器件(如空间光调制器)实现。Kner等^[1]^以11帧/s的成像速度跟踪了黑腹果蝇S2细胞微管蛋白和驱动蛋白动态。大致同时,Hirvonen等^[31]^展示了活COS-7细胞中线粒体运动的3D-SIM长时间成像。Shao等^[32]^随后对HeLa细胞中的线粒体和果蝇S2细胞中的微管进行了快速3D-SIM成像,速度为每步5 s,累计成像次数超过50次。

除了以上提到的,SIM超分辨成像技术在植物细胞结构的研究领域应用也颇为广泛,特别是胞间连丝的结构、植物细胞间运输和通讯的微观通道以及它们参与植物病毒的运输一直是最近研究的热门。相继出现的Hessian-SIM^[33]^和GI-SIM^[34]^分别从算法和照明两个角度,实现了对SIM成像的时间和空间分辨率优化。至此,科学家可以在长达1 h的时间内,对细胞内复杂结构如线粒体嵴,细胞内复杂生理过程如线粒体内质网相互作用进行更深入细致的研究。

### 2.2 STED

STED是第一个应用于细胞成像的远场超分辨率成像技术^[35]^。自1994年发明以来,已经在多种细胞生物学问题的研究中得到应用。

在神经细胞学的研究中,STED成像技术提供的纳米尺度分辨率,使研究者有可能进入前人难以触碰的领域^[8]^。将光稳定的染料ATTO-647N用抗体标记,Westphal等^[36]^借助STED研究了海马神经元活细胞的突触小泡,横向分辨率达到62 nm。研究者发现,突触束内突触小泡的活动高度受限,而束外的小泡则表现出更快的线性运动。这一差异可能体现了轴突的运动。

在细胞结构如脂膜、细胞骨架等研究中,STED技术同样具有灵活的应用。硅罗丹明具有光稳定性强、波长长的特点,它们可以很好地与市售775 nm的STED损耗激光结合。与较低波长激光相比,长波长激光的光毒性显著降低,因而硅罗丹明类染料在活细胞成像中特别受欢迎。采用与多烯紫杉醇融合的硅罗丹明SiR-tublin对细胞骨架进行STED成像,获得的分辨率相比于用微管结合蛋白融合蛋白标记提高了两倍。对于细胞内的微管组织中心——中心体,活细胞STED成像观察到其中心的中心粒是由9个三重微管组成的圆柱形结构。通过STED成像测得其直径为176 nm,相邻最大值之间的极角约为40°,与中心粒的9倍对称性一致,这与通过电子显微镜(EM)测得的结果基本一致,并且STED超分辨荧光成像还可以实现对活细胞的动态观察,这是电子显微镜无法做到的。SiR-actin是另一种基于硅罗丹明的探针,它可以实现对F-actin特异性标记。借助STED成像,研究者观察到SiR-actin染色的原代大鼠海马神经元轴突边缘呈环状结构,沿轴突轴均匀分布,周期为180 nm。随后,用SiR-tubulin进一步证实了这一周期性重复的细胞骨架结构是神经细胞的一般特征^[37]^。

细胞器之间相互作用及同一细胞器不同结构之间的动态变化通常发生在纳米尺度上,这时只有依赖超分辨显微成像才能实时观察到。两种及以上光谱有明显区分的染料配合使用的多色STED成像,在细胞动态研究大有可为。例如选取ATTO-590与硅罗丹明,配合蛋白标签可以实现不同细胞器相互作用或者同一细胞器不同结构的双色STED跟踪(见图3a)^[38]^。采用相同的荧光团与标记方式,科学家还发现小GTP酶ARF1参与高尔基体运输中细长管状载体的形成(见图3b,c)^[39]^。

**图3:**
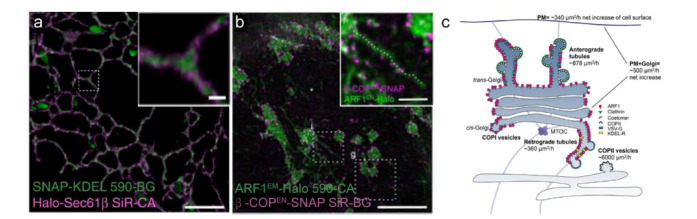
活细胞STED成像

### 2.3 SMLM

包括PALM、荧光光激活定位技术(photoactivated localization microscopy, FPALM)和STORM在内的单分子定位荧光显微成像技术与STED的根本区别在于这些技术的成像原理是基于单分子定位的。这些技术背后的基本原理是,如果收集到足够的光子,并且在衍射受限范围内没有其他类似发射的分子,单个分子的位置可以精确到1 nm或更好。这一概念由海森堡在20世纪30年代提出,并在20世纪80年代数学公式化,使得单分子跟踪研究成为可能^[40]^。Gelles等^[41]^对驱动蛋白修饰的小球的运动进行了成像,精度为1~2 nm。这一工作成为许多其他单分子成像研究的基础。

SMLM技术的突出优势在于空间分辨率。双色STORM呈现的固定哺乳动物细胞中的微管网络和网格状凹陷的组织,分辨率约为20 nm^[42]^(见图4a)。利用光学像散,Huang等^[43]^实现了三维(3D)STORM XY平面上20~30 nm的分辨率,*z*轴的分辨率可达到50 nm(见图4b)。利用多焦平面成像技术,Juette等^[44]^实现了三维FPALM成像,*xy*平面分辨率为30 nm,轴向分辨率为75 nm。

**图4:**
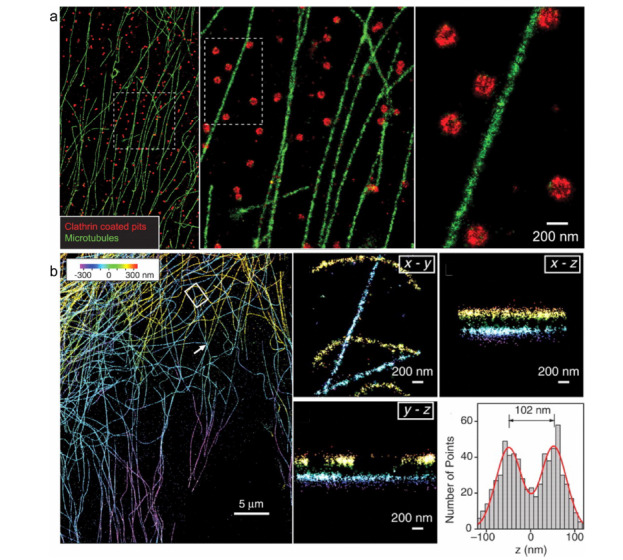
哺乳动物细胞BS-C-1中(a)微管和CCPs的双色STORM成像和(b) 3D-STORM成像

因此,活细胞SMLM用于跟踪高时空分辨率的生物过程和结构具有巨大的潜力,一个早期的例子是网格蛋白介导的转铁蛋白簇的内吞作用的表征^[45]^。在这些实验中,用Alexa 568标记转铁蛋白,并通过SNAP标签用Alexa 647(通过电穿孔引入)标记网格蛋白包被的凹坑。双色SMLM图像揭示了网格蛋白外壳和其中包裹的转铁蛋白在30 s内的形态变化。其中转铁蛋白单独的时间分辨率高达0.5 s,充分揭示了其集群的形成和内化过程。

具有光开关性质的膜特异性小分子荧光团使得观察复杂细胞器膜的结构动力学成为可能。例如,亲脂性膜染料DiI(1,1'-二十八烷基-3,3,3',3'-四甲基吲哚碳菁)被用于研究海马神经元质膜的结构和动力学。借助STORM成像获得的分辨率可以确定树突棘颈的宽度,而一系列高时间分辨率(10 s)成像的获得,为研究并揭示丝足和树突棘动力学和二者在不同区域的收缩和伸展提供了依据^[46]^。

随着更多适用于SMLM成像的染料出现,在更长时间跨度更小时间分辨率上获取活细胞的动态生理活动成为可能。HMSiR及HIDE是其中有所突破的典型。HMSiR的开关不需要高强度激光照射或额外试剂的添加,因此可以实现更长时间的SMLM成像。对该分子而言,激光强度可以降低到SMLM中标准荧光团所需的10%左右,大大减少了光漂白和光损伤。这允许在1 h的时间跨度内,以30 s的时间分辨率每隔10 min监测微管的运动^[47]^。进一步通过点击化学将HMSiR荧光团定位在不同的膜上,正如对其他HIDE探针所观察到的,亲脂性的膜环境将促进HMSiR分子的“开-关”平衡进一步向“关”的方向移动,使得密集标记的膜结构的成像成为可能。一个基于HMSiR的内质网HIDE探针观察到,细胞边缘分布着宽度为50 nm的内质网小管,这与以前的电镜数据一致^[48]^。

以上这些研究证明了SMLM作为活细胞动态研究的强大武器,但其应用范围的进一步扩大和延伸则需要更快的采集速度、更优的图像重构算法以及亮度更高的小分子染料。

## 3 总结和展望

包括SIM、STED及SMLM等在内的超分辨成像技术发展只有十多年的时间,但已经迅速展现出巨大的潜力^[10]^,并且空间分辨率的提高也正在缩小与电子显微镜之间的差距。几十年来,电子显微镜一直是亚细胞结构最精细细节成像的主流技术,但在样本动态活体实时成像上远不如荧光成像具有优势,然而包括共聚焦成像在内荧光显微镜技术在亚细胞结构的定位和鉴定中极大地受限于分辨率。到目前为止,超分辨成像技术的主要用途集中在生物应用上,用于研究小于200 nm结构变化的信息^[49]^。在这些应用中,超分辨成像技术通过详细的形态测量表征,以高灵敏度和高特异性检测和鉴定特定的生物分子。除了在结构和形态表征方面与生物分子检测、鉴定相结合外,超分辨成像技术也在迅速向相互作用图谱、多靶点检测和实时成像等领域扩展。在后一类应用中,超分辨成像技术尤以更灵活的样品染色,更高的标记效率,更快更简单的读数,更温和的样品制备程序而越来越具有优势。荧光蛋白和荧光染料是超分辨成像中主要使用的发色团,荧光蛋白具有标记准确和生物无毒的优势,但随着时空分辨提高所要求的荧光团更高亮度和稳定性的需求,化学工作者正更多地关注于新型荧光染料的开发。超分辨成像技术的革新带来了前所未见的微观世界的样貌,同时也激励化学工作者从机理上深刻了解荧光染料构效关系,从合成上开发新型超分辨荧光染料,与各领域研究工作者做好交叉,进一步借助超分辨成像技术探索微观世界。此外,提高对超分辨显微成像技术的认识,以及发现细胞中纳米级组织的前所未知的特征,也有助于研究者解决其他方法无法回答的全新的问题。
